# Vacuum-assisted breast biopsy: A comparison of 11-gauge and 8-gauge needles in benign breast disease

**DOI:** 10.1186/1477-7819-6-51

**Published:** 2008-05-19

**Authors:** Markus Hahn, Stella Okamgba, Peter Scheler, Klaus Freidel, Gerald Hoffmann, Bernhard Kraemer, Diethelm Wallwiener, Ute Krainick-Strobel

**Affiliations:** 1 Clinic for Obstetrics and Gynaecology, University of Tuebingen, Germany; 2 Clinic for Obstetrics and Gynaecology, St. Josefs-Hospital, Wiesbaden, Germany

## Abstract

**Background:**

Minimal invasive breast biopsy is standard care for the diagnosis of suspicious breast lesions. There are different vacuum biopsy (VB) systems in use. The aim of the study was to determine the differences between the 8-gauge and the 11-gauge needle with respect to a) diagnostic reliability, b) complication rate and c) subjective perception of pain when used for vacuum-assisted breast biopsy.

**Methods:**

Between 01/2000 and 09/2004, 923 patients at St. Josefs-Hospital Wiesbaden underwent VB using the Mammotome^® ^(Ethicon Endosurgery, Hamburg). Depending on preoperative detection, the procedure was performed under sonographic or mammographic guidance under local anaesthesia. All patients included in the study were followed up both clinically and using imaging techniques one week after the VB and a second time after a median of 41 months. Excisional biopsy on the ipsilateral breast was an exclusion criteria. Subjective pain scores were recorded on a scale of 0 – 10 (0 = no pain, 10 = unbearable pain). The mean age of the patients was 53 years (30 – 88).

**Results:**

123 patients were included in the study in total. 48 patients were biopsied with the 8-gauge needle and 75 with the 11-gauge needle. The use of the 8-gauge needle did not show any significant differences to the 11-gauge needle with regard to diagnostic reliability, complication rate and subjective perception of pain.

**Conclusion:**

Our data show that there are no relevant differences between the 8-gauge and 11-gauge needle when used for VB. Under sonographic guidance, the use of the 8-gauge needle is recommended for firm breast tissue due to its sharp scalpel point and especially for complete removal of benign lesions. We did not find any advantages in the use of the larger 8-gauge needle compared to the 11-gauge needle in the mammography setting. The utilisation costs of the 8-gauge needle are somewhat higher.

## Background

Vacuum-assisted breast biopsy (VB) under sonographic [1-4] and mammographic [5,6] control is recognised as an established method of minimal invasive tissue extraction. VB is recommended as a diagnostic method in the S3-guidelines for the early recognition and diagnosis of breast cancer [7]. In contrast to open biopsy, the technique represents a minimal invasive intervention for the clarification of unclear focal lesions in the breast, and it can furthermore be used for the diagnostic-therapeutic complete removal of benign lesions [8-12]. In contrast to fine needle aspiration and minimal invasive high speed core needle biopsy with the 14-gauge needle, the diagnosis of the smallest solid lesions of the breast as well as microcalcification are certainly possible with this technique. VB consequently closes the gap between open biopsy and the small calibre minimal invasive procedures and completes the spectrum of techniques.

**Figure 1:**
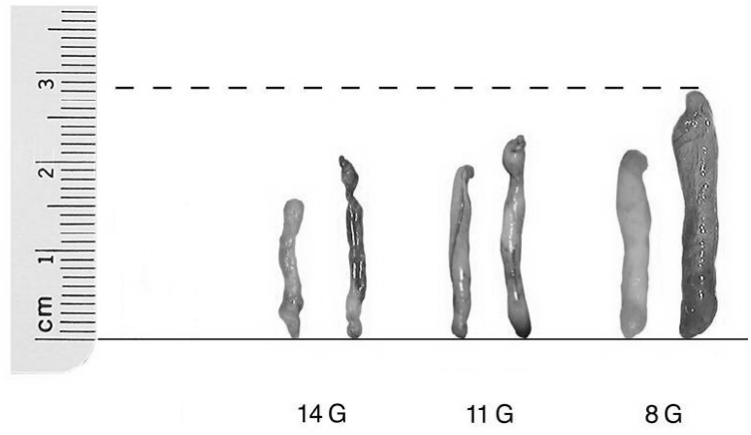
The 14, 11 and 8 gauge tissue cylinders.

**Figure 2:**
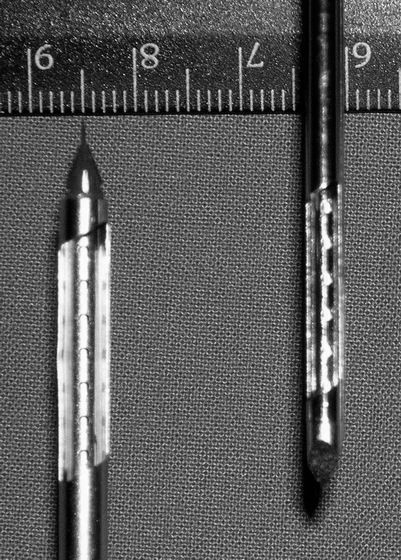
The 11 and 8 gauge needle.

**Figure 3:**
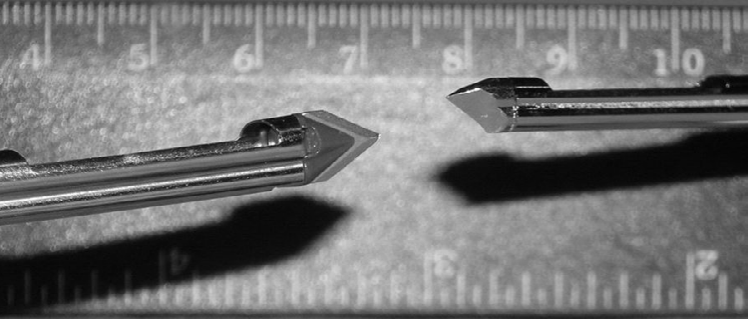
11 and 8 gauge needle (sideview).

The mammographic VB was first clinically tested by Steve Parker in 1994 (14-gauge). Since 1996, the 11-gauge needle has been routinely used in the Mammotome^®^-System in clinical practice. The Mammotome^®^-vacuum biopsy system was completed with addition of the 8-gauge needle in 2001. A further VB system, the Vacora^®^, [13] works with a 10-gauge needle diameter. Summaries of the dimensions of the various biopsy needles and the tissue cylinders (figure 1) are shown in tables 1 and 2 (figure 2 and 3).

**Table 1:** External measurements of the needles and incision length on the skin

	Width in mm	Height in mm	Incision length in mm
11G	3,1	4,6	4
10G	4,0	4,0	4,5
8G	4,3	6,0	5

**Table 2:** Dimensions of the tissue cylinder dependent on the needle window size

	Length in mm	Weight in mg	Diameter in mm	Volume in mm^3^
11G	19,4	100	2,16	71
10G	19	170	3,4	170
8G	23	300	3,35	203

The aim of this study was to evaluate the differences between the 11-gauge and 8-gauge needles of the Mammotome^®^-System with regard to a) diagnostic reliability, b) complication rate and c) subjective perception of pain both immediately postoperatively and on follow-up.

## Methods

### Patient selection

Between 01/2000 and 09/2004, 923 patients at St. Josefs-Hospital Wiesbaden underwent VB using the Mammotome^® ^(Ethicon Endosurgery, Hamburg). 123 patients could be included in the study. Inclusion criteria were benign histology and complete follow up (inspection, palpation, mammogram and sonogram). An open biopsy was not performed after extirpation by VB.

Operative interferences on the ipsilateral side were exclusion criteria. Consequently, only patients with benign histology were followed up in the scope of this study, since patients with malignant histology needed to be supported with operative treatment. The VB was performed for lesions classified as BI-RADS^© ^3, 4 and 5. Imaging control was performed using the technique which gave the most accurate representation of the findings. 48 patients (39%) were biopsied with the 8-gauge needle and 75 (61%) with the 11-gauge needle.

The mean patient age was 53 (30–88) years. The median follow-up period was 41 (5 – 64) months.

### Biopsy technique

All biopsies were performed using VB-equipment from Ethicon Breast Care Mammotome^®^. A table from Fischer Imaging^® ^was used for stereotactic procedures. Ultrasound equipment HDI 5000 from ATL^® ^was used for sonographic biopsies in Sono-CT mode. All biopsies were performed as an out-patient procedure. 20 ml of Prilocaine 1% with epinephrine 1:200000 was used as local anaesthetic. It was applied subcutaneously and not peritumorally in order to not interfere with the imaging. Further drugs were not administered. The biopsy site was compressed postoperatively until bleeding ceased. The incision site was covered with a Steri-Strip plaster. Finally, a thorax pressure bandage was applied for 24 hours.

### Follow-up

Haematomas were differentiated according to need for revision and superficial cutaneous development. Superficial cutaneous haematomas were recorded according to persistence in days.

Infections requiring antibiotic treatment as well as cutaneous scar formation in the incision area were also evaluated. In order to evaluate patient acceptance, all patients were questioned about their subjective perception of pain immediately postoperatively, 1 week postoperatively and at the last follow-up appointment. A pain scale from 0 (no pain) to 10 (unbearable pain) was used for this.

### Statistical analysis

Data was collected using Microsoft Access, and the statistical analysis was carried out using STATISTICA^®^. Chi squared tests were used. Whether or not the perception of pain differed between the patients and over the three measuring times was tested with the help of a multi-variant analysis (MANOVA). Post hoc comparisons were statistically confirmed using a t-test. The description of mean values was completed using standard deviations (MW ± SD).

## Results

A correct diagnosis could be made for all 123 patients using VB. No repeat biopsies had to be performed. No biopsies were abandoned during the procedure. Out of the 123 biopsies performed, a total of 46 fibroadenomas, 18 cases of sclerosing adenosis, and 38 cases of fibrocystic mastopathy, 11 cases of scarring following a previous surgical procedure, 8 papillomas and 2 cases of other benign histology were diagnosed. All 8 papillomas which were included in the study showed no signs of atypia and were completely removed under sonographic imaging. Therefore open biopsy was not performed after recommendation in an interdisciplinary tumour conference. No residues were found in any of these cases at the follow-up examination. A list of the histological results stratified according to needle size is shown in table 3.

**Table 3:** Histological findings after biopsy

Needle size	Fibroadenoma	Sclerosing adenosis	Fibrocystic mastopathy	Scar tissue	Papilloma	Other
11G	29	12	23	5	4	2
8G	17	6	15	6	4	0
Total	46	18	38	11	8	2

It should be noted that only benign lesions which did not need to be reoperated could be included in the study. Follow-up would otherwise not have been possible, as an operation would have followed. No malignancies were diagnosed at the follow-up examination. Similarly, no residues requiring biopsies were found.

48 patients (39%) underwent biopsy using the 8-gauge needle and 75 (61%) with the 11-gauge needle. An overview of the results is shown in table 4.

**Table 4:** Results

	**8 g**	**11 g**
Representative removal	20 (41, 7%)	40 (53, 3%)
Complete removal	28 (58, 3%)	35 (46, 7%)
Haematomas requiring revision	0	0
Cutaneous haematomas	36 (75%)	57 (76%)
Mean duration of haematomas in days	13	10
Infection requiring antibiotics	0	0
Noticeable external scar formation	5 (10, 4%)	8 (10, 7%)
Mean pain score immediately postoperatively	3,6	3,2
Mean pain score 1 week postoperatively	0,7	0,6
Mean pain score in follow up	0,1	0,3
Max. lesion diameter in mm	50	36
Min. lesion diameter in mm	4	2
Mean lesion diameter in mm	16	15

### Diagnostic reliability

The mean diameter of the lesions using the 8-gauge needle was 16 mm (4–50 mm), and with the 11-gauge needle was 15 mm (2–36 mm). Out of the 48 biopsies performed with the 8-gauge needle, the lesion was completely removed in 28 (58%) cases. A representative biopsy was performed in 20 (42%) cases. Using the 11-gauge needle, complete removal was achieved in 35 (47%) cases, and a representative removal in 40 (53%) cases in this group.

There was no significant difference (p = 0.2) in diagnostic reliability between the 8-gauge and 11-gauge needle.

### Haematomas

Haematomas requiring revision did not occur in either the 8-gauge or the 11-gauge group. Superficial cutaneous haematomas were noticed in 36 (75%) patients after the 8-gauge biopsy and 57 (76%) patients after 11-gauge biopsy. The mean persistence of the haematomas in the 8-gauge group was 13 (2–43) days and 10 (2–56 days) in the 11-gauge group.

### Scars

Noticeable external scars, none of which were aesthetically unacceptable or required correction, occurred 5 times in the 8-gauge group and 8 times in the 11-gauge group. No significant difference was seen here (p = 0.6).

### Perception of pain

The groups did not differ significantly from one another with regard to subjective pain perception (p = ns). Pain perception was also similar when the respective questioning times were compared (t-Test, p > 0.06). The mean subjective pain score immediately postoperatively was 3.5 ± 2.6 in the 8-gauge group and 3.0 ± 2.7 in the 11-gauge group. One week postoperatively, the mean pain score was 0.9 ± 1.4 in the 8-gauge group and 0.8 ± 1.5 in the 11-gauge group. At the last follow-up appointment, the mean pain score was 0.0 ± 0.3 in the 8-gauge group and 0.1 ± 0.5 in the 11-gauge group. The perception of pain abated comparably in both patients groups postoperatively (MANOVA F_2;228 _= 140.57, p < 0.001; the difference between the groups over the questioning times was not significant (MANOVA F_2;228 _= 1.13, p = 0.33). However, the pain did not only reduce in the week following the procedure (t-Test, p < 0.001), but also up to the last follow-up appointment and was once again significant in both groups (t-Test, p < 0.01).

## Discussion

The Mammotome^®^-System has been in clinical use with the 11-gauge VB needle since 1996. In 2001, the system was completed by the addition of the 8-gauge needle.

The 11G or 8G needle was chosen depending on the size of the lesion. Use of the 8G needle was recommended from a size of 15 mm; however the ultimate choice was made by the surgeon. Since the data were already collected at the start of 01/2000, i.e., before publications such as, for example, the consensus recommendation from Krainick-Strobel et al., no reference to literature recommendations concerning needle choice could be made.

The first impressions of 8-gauge needle with respect to the cylinder quality as well as the tissue volume withdrawn per examination time are convincingly good (figure 1). Even the fragmentation of the tissue cylinder seen with the 11-gauge needle is rarely seen with the 8-gauge needle.

The increased trauma to the breast tissue suspected initially has been neither subjectively nor objectively confirmed. In contrast, the shortened procedure duration, which comes as a result of a larger tissue volume being removed per cylinder, is an advantage for both the patient and clinician [14]. This evaluation is similar to that of Diebold et al. [15].

The results of this study show no significant differences between the two needle sizes. A precise analysis of the procedure duration was not performed and should be undertaken in further work. The consensus recommendation for stereotactic VB related to the 11-gauge needle [5]. This is due to the fact that the 8-gauge needle was not on the market at the time of the consensus finding. The consensus recommendation for VB under sonographic guidance differentiated, however, between an 8-gauge and 11-gauge needle [2].

According to the consensus recommendation for the sonographic application of VB [2] and in the light of the data from Krainick et al. [16] and Hahn et al. [8], it is recommended that fibroadenomas with a maximum diameter of up to 18 mm are removed using the 8-gauge needle and those with a maximum diameter of up to 11 mm with an 11-gauge needle. However, the assumption that a larger tissue volume can be removed using a larger needle is only partly correct. In fact, the maximum tissue volume which can be removed is limited by bleeding, the size of the breast and the site of the lesion (e.g. close to the skin surface) [17].

In 8 cases the histology result showed papilloma without atypia. In all 8 cases the lesions were completely removed under sonographic imaging. In all cases the patients declined open biopsy. It should be pointed out that it is controversial to follow papillomas following core biopsy alone.

Based on our data and experience, the use of the 8-gauge needle for very firm glandular tissue is sensible especially under sonographic imaging. Exact guidance of the needle is easier to perform with the 8-gauge needle than with the 11-gauge needle due to the scalpel point on the 8-gauge needle.

The 11-gauge needle seems to be sufficient for stereotactic applications. In individual cases, where extensive tissue removal is necessary or in the case of very firm glandular tissue, the 8-gauge needle can also be helpful under stereotactic guidance.

It ultimately remains an individual decision as to which needle size the surgeon chooses [18]. A rough orientation guide for the choice of needle size is given in table 5.

**Table 5:** Recommended needle size – indications

Diagnosis of microcalcification (stereotactic)	**11G**
Focal lesions without microcalcification (stereotactic)	**8G**
Sonographic removal of intraductal, intracystic lesions	**8G**
Sonographic removal of fibroadenomas up to 11 mm diameter	**11G**
Sonographic removal of fibroadenomas up to 18 mm diameter	**8G**
Re-biopsy for failed correlation of suspected diagnosis and histology	**8G**
Sonographic removal of suspicious lesions smaller than 5 mm	**11G**

The 8-gauge and 11-gauge needles vary in price, the 8-gauge needle with its dependence on imaging control being more expensive than the 11-gauge needle.

Just as with all other new methods, the vacuum breast biopsy must be further evaluated in clinical use with a higher number of patients.

## Conclusion

Our data show that there are no relevant differences between the 8-gauge and 11-gauge needle when used for VB. Under sonographic guidance the use of the 8-gauge needle is recommended for firm breast tissue due to the sharp scalpel point, and especially for complete removal of benign lesions. We did not find any advantages in the use of the larger 8-gauge needle compared to the 11-gauge needle in the mammography setting. The utilisation costs of the 8-gauge needle are somewhat higher.

## Competing interests

The authors declare that they have no competing interests.

## Authors' contributions

MH and PS were the surgeons who performed all biopsies. MH and SO designed the current study and collected the data. MH and KF performed all data analyses. GH, BK, DW and UK–S edited the manuscript. All authors approved the final version of the manuscript.
